# Suprachoroidal VIsco-bucKlING versus gas tamponade for the treatment of rhegmatogenous retinal detachment (VIKING): study protocol for a multi-centre, randomised, controlled feasibility study

**DOI:** 10.1186/s40814-026-01787-w

**Published:** 2026-03-17

**Authors:** Shohista Saidkasimova, Chan Ning Lee, George S. P. Murphy, Timothy L. Jackson

**Affiliations:** 1 King’s Ophthalmology Research Unit, Department of Ophthalmology, King’s College Hospital, London, UK; 2 Faculty of Life Science and Medicine, King’s College London, London, UK; 3 Tennent Institute of Ophthalmology, Gartnavel General Hospital, Glasgow, G12 0YN UK

**Keywords:** Retinal detachment, Surgery, Vitrectomy, Suprachoroidal viscobuckling, Gas tamponade, Recovery, Feasibility, Randomised controlled trial

## Abstract

**Background:**

Rhegmatogenous retinal detachment (RRD) is a potentially blinding condition that affects ~ 1 in 10,000 adults per year. Retinal detachment surgery usually involves internal reapposition of the retina using the injection of a gas tamponade into the vitreous cavity. However, this has downsides including delayed visual recovery, the need to posture postoperatively, limiting fundal view during postoperative monitoring, and the risk of raised intraocular pressure. We examine a novel surgical variation, using a temporary suprachoroidal viscobuckle (SCVB) instead of gas. SCVB involves the controlled injection of high viscosity viscoelastic through an external scleral incision into the suprachoroidal space to create an indent under a causative retinal break. The sodium hyaluronate viscoelastic is resorbed over 2–3 weeks, long enough for intraoperative laser retinopexy to cause chorioretinal adhesion. The primary objective of this study is to establish if it is feasible to recruit, retain, and evaluate patients with RRD into a larger randomised controlled trial of SCVB. Secondary objectives are to make a preliminary assessment of safety and efficacy.

**Methods:**

This multicentre, randomised, controlled, two-group, unmasked feasibility trial recruits participants with primary RRD and a break or breaks within 1 clock hour. The CE-marked sodium hyaluronate viscobuckle is created during otherwise standard pars plana vitrectomy RRD surgery. Feasibility outcomes include screen failure rate, rate of recruitment, protocol compliance, and participant retention. The main efficacy outcome is retinal attachment with no intravitreal tamponade present and no subretinal fluid which could spread, without the need for further surgery, at week 12. The key secondary efficacy outcome is Early Treatment of Diabetic Retinopathy Study best-corrected visual acuity at week 24. Safety is recorded through adverse events and serious adverse events.

**Discussion:**

SCVB has multiple potential advantages for patients. This study will help determine if a definitive study of SCVB is justified and deliverable and inform its design.

**Trial registration:**

ClinicalTrials.gov Identifier: NCT04557527, registered 09 September 2020.

**Supplementary Information:**

The online version contains supplementary material available at 10.1186/s40814-026-01787-w.

## Background {6a, 6b}

Rhegmatogenous retinal detachment (RRD) is a potentially blinding condition that occurs when a break in the neurosensory retina allows vitreous fluid to pass into the subretinal space, lifting the neuroretina off the underlying retinal pigment epithelium (RPE). This typically causes a corresponding visual field defect that expands over time to involve the fovea, with loss of central vision. RRD affects ~ 1 in 10,000 adults per year in the United Kingdom (UK) and is the most common indication for retinal surgery [1–3]. Most RRDs require surgical repair, with the most common operation in the UK being vitrectomy, cryotherapy (cryo), or laser retinopexy and intravitreal gas tamponade; the next most frequent is scleral buckling, then combined vitrectomy with scleral buckling [2]. The primary reattachment rate following surgery has been reported as 80–90% [1, 2, 4].

The use of intravitreal expansile gases in RRD repair is burdensome for patients due to the need for head posturing (to maximise tamponade effect across the break) and severely reduces vision whilst the gas fills the eye. The gases take about 2–8 weeks to absorb; during this time, patients cannot fly, are advised not to drive, and must not have general anaesthesia using nitrous oxide which can cause gas expansion. Gases may increase the likelihood of post-vitrectomy cataract and postoperative intraocular pressure (IOP) elevation. Gases obscure the fundal view, making post-operative retinal examination and monitoring difficult.

Scleral buckling is one of the oldest recognised surgical procedures for the treatment of RRD [5]. It involves the suturing of a silicone explant onto the sclera to produce an indentation over the detached retina and retinal breaks [6]. This promotes retinal reattachment when combined with retinopexy to seal the break via a chorioretinal adhesion. Difficult surgical techniques, prolonged recovery and associated complications, i.e. injury to the extraocular muscles and diplopia, distortion of the globe and refractive change, ocular ischaemic syndrome, and exposure of the implant, have meant that surgeons have generally shifted away from this technique [2].

A technique that combines the advantages of vitrectomy and scleral buckling, without the burden of gas tamponade, could materially impact how vitreoretinal surgeons undertake the most commonly performed retinal operation [3].

Previous attempts to achieve gas-tamponade free retinal reattachment surgery have included the use of fibrin glue and meticulous laser [7–10], but neither has had widespread adoption.

This study investigates a variation to vitrectomy surgery, wherein a temporary suprachoroidal viscobuckle (SCVB) is used instead of gas tamponade. SCVB is a new technique that involves the controlled injection of viscoelastic into the suprachoroidal space to create an indent similar to scleral buckling under direct visualisation. High viscosity sodium hyaluronate ophthalmic viscosurgical device (OVD) has been safely used in the eye for this purpose and has a mean elimination time of 2 weeks, long enough for retinopexy to cause a chorioretinal adhesion [11]. Although the injection of sodium hyaluronate OVD into the suprachoroidal space to create a buckle effect is novel, it has a long history of intraocular use and is widely used as a surgical device in standard cataract surgery [12].

Compared to conventional scleral buckling with a silicone implant, SCVB offers a minimally invasive approach, with no manipulation of the extra-ocular muscles and potentially quicker recovery. Compared to vitrectomy, it avoids all the downsides of intravitreal gas tamponade.

Retrospective case series showeed good success and low complication rates with SCVB (REFS) [13–16]. Four case series of 14 to 62 patients (138 in total) report a primary retinal reattachment success rate between 92 and 100% [13–16], similar to reported reattachment rates of 80–90% [1, 2] with standard RRD surgical techniques. Complications were reported to be rare [14–17] and included single incidences of bleeding from the sclerotomy site, localised retinal or choroidal haemorrhage, retinal or choroidal trauma, transient elevation of IOP; none of which required further intervention.

To realise the potential patient benefits of SCVB surgery, we first need to establish that SCVB has an anatomic and visual outcome that is no worse than standard vitrectomy. That is likely to require a large, randomised, non-inferiority trial. Such a study will be expensive and is unlikely to attract Industry funding. We therefore need a robust feasibility study before we are able to apply to a government or charitable funder to undertake a definitive study.

The suprachoroidal viscobuckling versus gas tamponade for the treatment of rhegmatogenous retinal detachment (VIKING) trial aims to establish if a pivotal randomised controlled trial (RCT) is justified and deliverable and to inform its design.

### Trial design {8}

VIKING is a multicentre, two-group, randomised, if the surgical device is non-masked device feasibility trial.

### Objectives {7}

This study is designed to determine if a larger, efficacy study is justified and deliverable, and to inform the design of any such study. A subsequent study is likely to have a non-inferiority design, wherein the primary hypothesis is that the patient advantages of SCVB (no gas tamponade and possibly fewer cataracts) can be realised without a worse anatomic or visual outcome.

The primary objective is to establish if it is feasible to recruit, retain, and evaluate patients with RD into a larger randomised controlled trial of SCVB.

Secondary objectives are to make a preliminary assessment of the safety and efficacy of SCVB.

## Methods: participants, intervention, outcomes

VIKING is reported in line with the CONSORT 2010 extended guidelines for randomised pilot and feasibility trials [18], and the protocol has been developed in line with SPIRIT guidelines [19]. A SPIRIT figure is included in Table 3 in the Appendix (as the schedule of assessments), and the SPIRIT checklist is included in the supplementary appendix (Appendix A). The trial sponsors are King's College London and King's College Hospital (Clinical Co-Sponsor).

### Setting {9}

VIKING aims to recruit 50 participants over 2 years from six NHS hospitals. A multi-centre approach will improve the likelihood of recruiting to target in the desired time frame, but also to ensure participants are drawn from a broader population than afforded by a single study site.

### Consent {11b, 26a}

Written informed consent will be obtained by a site’s principal investigator (PI) or delegated sub-investigator (SI) before any study procedures are undertaken, including screening. Patients will be recruited from the day of presentation and will be given 24–72 h to make the decision whilst awaiting urgent surgery. The short consent period is required due to the need for urgent surgery in most cases. Nonetheless, we anticipate that most patients will be able to take the information sheet home overnight, with time to discuss its contents with friends and family if desired. They can hold off joining the study right up to the point of surgery, to give as much time as possible to consider the options. Conversely, participants who have already enrolled will be made aware that they can withdraw from the study right up until the point of surgery and their consent will be reconfirmed during the pre-operative ward round (they can of course withdraw after surgery, but by then they may have already received SCVB). RRD surgery has an appreciable failure rate of about 10–20% [1, 20]. It is possible that patients may attribute any surgical complications or surgical failure to SCVB when these were anyway inherent to RRD surgery. Careful explanation will be provided to communicate that SCVB may not change the surgical outcome but is aimed at improving the recovery time after surgery. The general risks of surgery will be detailed in the patient information sheet, separately to those thought possible due to the altered surgical step (SCVB instead of gas).

#### Eligibility criteria

##### Inclusion criteria

Males and females aged at least 18 years (including females of childbearing age) requiring pars plana vitrectomy for the treatment of primary RRD caused by a single break or multiple breaks within one clock hour. The final determination of qualifying breaks is made at the time of surgery following 360-degree internal indented search using a wide-angle viewing system.

##### Exclusion criteria

General:

- Hypersensitivity to sodium hyaluronate OVD.
- Participation in another interventional study within 8 weeks of enrolment or planned to occur during this study.
- Bleeding disorders or the use of anticoagulants (such as warfarin, rivaroxaban) or dual anti-platelet drugs such as aspirin with clopidogrel. Monotherapy with low dose (≤ 100 mg) aspirin is permitted, and if clinically appropriate this should be stopped prior to surgery and recommenced only after satisfactory day 1 postoperative review.
- Unwilling, unable, or unlikely to return for scheduled follow-up for the duration of the trial.
- Any other condition that, in the opinion of the investigator, would prevent the participant from granting informed consent or complying with the protocol, such as dementia, mental illness, or serious systemic medical disease.

Study eye:

- Presence of proliferative vitreoretinopathy (PVR) or any tractional RD.
- Previous vitreoretinal surgery, open-globe injury, or endophthalmitis.
- Aphakia.
- Congenital cataract.
- Previous or current suprachoroidal haemorrhage.
- Congenital or acquired ocular, orbital or periocular abnormality that, in the opinion of the attending vitreoretinal surgeon, would preclude the safe delivery of the OVD into the suprachoroidal space.
- Presence of other ocular comorbidity that, in the opinion of the investigator, is likely to prevent an accurate assessment of retinal attachment.
- Current intraocular inflammation other than mild cellular activity that is thought to be secondary to RRD.
- Current ocular or periocular infection other than blepharitis.

The reasons for ineligibility or withdrawal will be recorded and described.

#### Intervention

This RCT is comprised of 2 arms:

1. Control arm—participants undergo standard vitrectomy, cryo or laser retinopexy, and gas procedure. The choice of gas (SF_6_, C_2_F_6_, or C_3_F_8_) is determined by the surgeon’s preference and position of the break. Participants are advised to posture postoperatively, dependent on the position of the break.
2. Study arm—vitrectomy, SCVB, and laser retinopexy procedure.

The SCVB surgical technique involves a small incision in the sclera, 5–6 mm behind the limbus in the clock hour of the break, to access the suprachoroidal space. A small amount of viscoelastic is injected to bluntly dissect choroid from the sclera. These structures are opposed rather than attached to each other; therefore, very little resistance is felt when opening this potential space. The SCVB is created under direct visualisation by the surgeon injecting the viscoelastic while adjusting the direction of the cannula directly under the break. The height of the indent should be sufficient to cover the perimeter of the break. The breaks are treated with laser retinopexy after creation of the indent. Air tamponade is allowed if the surgeon deems it necessary, but that does not mandate posturing and will absorb much quicker than gas tamponade. The suprachoroidal buckling procedure may be abandoned in favour of standard of care if a complication took place, i.e. clinically significant choroidal or retinal injury or significant haemorrhage, at the surgeon’s discretion.

The trial commenced using HEALON5™ PRO OVD (Johnson & Johnson Surgical Vision, Inc., Wokingham, UK). The Healon family of viscoelastics has been in use for over two decades, and we estimate that it has been used in millions of eye operations. We know of no clinical concerns about biocompatibility or toxicity. The HEALON5™ PRO OVD used in this trial is used within its marketing authorisation: ‘Sodium hyaluronate OVD can also be used to efficiently separate and control ocular tissues. It is not designed to have any pharmacological effect’ [21]. The protocol was amended to allow the use of two very similar products, OphteisBio (Rayner, Worthing, UK) and NuViscTM PRO (BVI Medical, Elland, UK), also used within their marketing authorisation.

### Concomitant care {11d}

All concomitant care determined necessary by site investigators is permitted for this study and will be recorded in case report forms. The time to and proportion of participants who experience a re-detachment in their study eye is an important outcome for this study; however, participants will not undergo repeat SCVB surgery if they experience a re-detachment. Standard of care vitrectomy surgery will be offered instead.

### Randomisation {16a, 16b, 16c}

Randomisation and allocation are performed in the operating theatre using the centralised web-based Castor electronic data capture (EDC) system (Amsterdam, Netherlands), after the internal search of the eye and exclusion of additional breaks in the detached retina. Those who were found to have additional breaks will be documented as screen failures. The PI or delegated SI will undertake randomisation, and all have been allocated logins to the VIKING study EDC. Allocation occurs on the web portal immediately and is 1:1 to either SCVB or comparator with no randomisation stratifiers.

### Masking {17a}

No masking procedures will be undertaken.

### Schedule of visits {13}

**Fig. 1:**
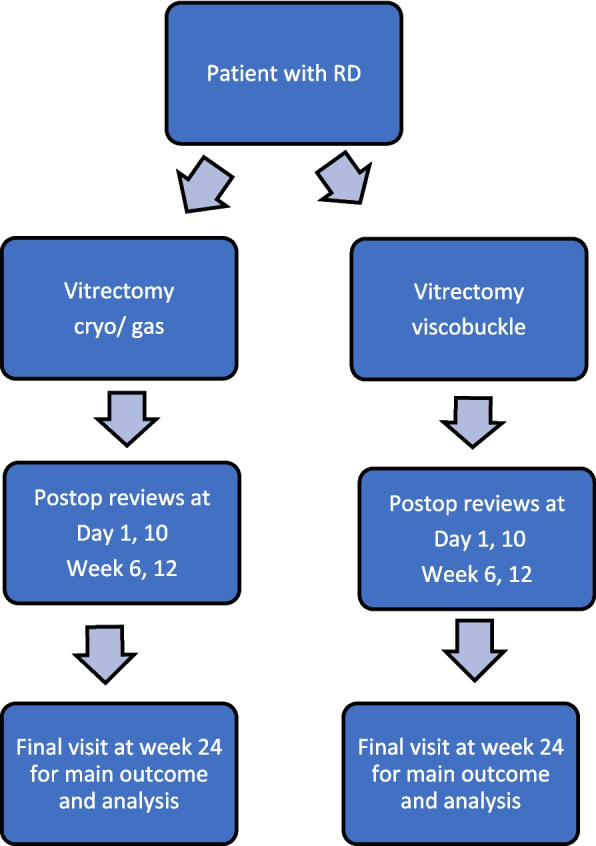
Flow chart demonstrating the participant pathway

The patient flow is shown in Fig. 1, with the schedule of procedures by visit in Table 1.

**Table 1:** Schedule of assessments {13}

	Baseline	Surgery	Day 1	Day 10	Week 6	Week 12	Week 24
Visit number	1	2	3	4	5	6	7
Visit window (days)	Day −21 to 0	NA	0	± 3 days	± 14 days	± 14 days	± 30 days
Screening and consent	R						
Demographics and history	R						
Best-corrected visual acuity (ETDRS)	R						R
Snellen visual acuity			x	x	x	x	
Slit lamp examination	x		x	x	x	x	x
Intraocular pressure	x		x	x	x	x	x
Lens grading (phakic eyes only)	R		*	*	*	*	R
Vitrectomy and retinopexy		X					
*Intervention group:* Suprachoroidal hyaluronic acid		R					
*Control group:* Gas tamponade		X					
Conmeds	R	R	R	R	R	R	R
Adverse events	R	R	R	R	R	R	R

*R* research visit, *X *standard of care assessment

*phakic eyes only

Participants will be treated at baseline and followed up in accordance with typical standard of care, on day 1, day 10, week 5, 12, and week 24.

### Recruitment {15}

Participants will be recruited from NHS outpatient clinics, having been referred from general practitioner (GP) surgeries, community optometrists and other ophthalmologists. The estimated duration of recruitment is 24 months.

Participants will be recruited from the day of presentation. The examining surgeon and/or research nurse will identify and approach patients for consent.

### Statistical analysis {14, 20a, 20b, 20c}

VIKING is not powered to test an efficacy-based hypothesis and no interim analysis is planned. The results will instead be descriptive, without any statistical comparisons. As noted in the section above, this study is not designed to test a hypothesis, but rather to inform a future study. We selected 25 participants for each arm, as that will give an estimate of visual acuity variance and may start to expose any difference in anatomic success rate [22, 23].

A total of 50 patients will provide a reasonable estimate of screening success, recruitment rate, retention, the ability to evaluate patients, attrition and time to ‘stress test’ the protocol, addressing the main feasibility outcomes of the study. With a sample size of 50 participants, we will be able to estimate the proportion of participants with an evaluable primary outcome of 80% with a 95% confidence interval of ± 11%.

From a feasibility perspective, we anticipate that a pivotal trial would be deliverable if the criteria detailed in Table 2 were met.

We recognise that the decision to proceed with a subsequent pivotal trial depends on an appreciation of the totality of the results, not just the feasibility metrics, including the apparent risks, benefits, estimated effect size, minimum clinically important difference, future unmet need, suitable funding streams, input from patient focus groups, amongst other factors (Table 2).

**Table 2:** Feasibility thresholds for a future study

Feasibility metric	No-go	Protocol modifications	Proceed without change
Recruitment (patients per month per site)	< 0.1*	0.1–0.3	> 0.3
Evaluable primary outcome (combining attrition and otherwise missing primary outcome data)	80%	81–88%	> 88%

*A slowly recruiting trial may be justified if the sample size is deliverable, and the clinical impact warrants an extended recruitment timeline and/or a large number of sites

A description of baseline demographics and ocular characteristics will be provided by group to include age, sex, best-corrected visual acuity (BCVA), laterality, lens status, location, and number of retinal breaks, and RD location.

The efficacy population will be ‘intent-to-treat’, including all randomised participants, but a ‘per protocol’ analysis will include all those randomised to SCVB who received it, and all those randomised to standard vitrectomy who received it.

For continuous efficacy variables, we plan to report the mean and standard deviation, unless the data appear skewed, in which case we will consider reporting the median and interquartile range. For categorical values, we will report proportions, with 95% confidence intervals.

In addition to the main BCVA results, BCVA will be reported in the subgroups of eyes that are phakic and pseudophakic, and those with macular-on and macular-off RDs, since post-vitrectomy cataract and macular status can materially influence BCVA outcomes.

### Patient and public involvement

We have consulted members of the public and patients who have recently had RD treatment in designing the study and patient information sheet. VIKING offers an intrinsically patient-focused modification but will also benefit society if patients can return to work sooner and not be as dependent on others during their convalescence. This small study is designed to inform the next phase of the study if that is shown to be feasible. Thus, we will consider inviting participants exiting this study to provide qualitative feedback on their experience with surgery and the trial and invite a selection of interested participants to join a Patient Public Involvement group for any next study.

### Data handling and management {18a, 19, 27}

Participant clinical outcome data will be pseudonymised at the point of collection using the participant’s allocated study identification, entered at site level on paper case report forms or into the site’s electronic medical records systems, and transcribed onto the VIKING study Castor EDC system. Paper case report forms will be kept at sites within locked, secure storage areas, whilst electronic data is secured on the Castor EDC server which is password-protected.

### Adverse events and incident reporting {22}

The safety population will be all participants who underwent RD surgery. All adverse events (AE), Adverse Device Effect (ADE), Serious Adverse Event (SAE), and Serious Adverse Device Effects (SADE) will be recorded. Severity will be classified as mild, moderate, and severe; and causality as definite, probable, possible, unlikely, not related, and not assessable; and expectedness as expected and unexpected as determined by the site investigator. Although not an SAE, any Important Medical Events (IME) and unplanned pregnancy will also be reported via the SAE reporting system.

All SAE/SADE/UADEs will be reported to the Sponsor’s R&I office within one working day of the investigator team becoming aware of them. Reports of related and unexpected SAEs will be submitted to the Research Ethics Committee (REC) within 15 days of the Chief Investigator becoming aware of the event, using the SAE/SADE report form. Events will be followed up until resolution, and any appropriate further information will be sent by the research team in a timely manner.

### Frequency and plans for auditing trial conduct {23}

Monitoring of this study is delegated to CI and co-CI and includes adherence to the study protocol, procedures for consent, and data quality. The CI will inform the sponsor should there be any concerns arising over monitoring activities or procedures.

### Protocol amendments {25}

All substantial (such as changes to eligibility criteria, outcomes, analyses) and non-substantial amendments to all REC-approved documents will be submitted to the REC for health research authority (HRA) approval by the CI or co-CI. These will be reflected in subsequent update publications to the trial protocol.

## Discussion

RRD surgery is mostly an effective treatment. However, it imposes a material burden on patients due to gas tamponade. A surgical technique that avoids (or minimises) gas tamponade would speed patient recovery, avoid postoperative posture, and may mitigate some of the clinical sequelae of gas tamponade. This would be the first RCT on the topic and may lead to a pivotal trial that could benefit patients undergoing the most commonly performed vitreoretinal operation.

### Trial status

The trial was completed on the 1 June 2025 and results will be published in 2026. Trial is registered on clinicaltrials.gov on the 09 September 2020 registration: NCT04557527. https://clinicaltrials.gov/study/NCT04557527.

## Supplementary Information

Supplementary Material 1.

Supplementary Material 2.

Supplementary Material 3.

## Data Availability

The lead sponsor (King’s College London) and clinical co-sponsor (King’s College Hospital) own and control the trial data. The CI and co-CI will have access to the final trial dataset for analysis. All data generated during this study and its supplementary information files, including amendments, will be included in a published article upon completion of the study. The datasets generated and/or analysed during the current study are not publicly available due to data protection requirements.

## Appendix

### Administrative information

The numbers in curly brackets in this protocol refer to SPIRIT checklist item numbers. The order of the items has been modified to group similar items (see http://www.equator-network.org/reporting-guidelines/spirit-2013-statement-defining-standard-protocol-items-for-clinical-trials/). A full SPIRIT checklist is available as a supplementary appendix (Appendix A).

**Table 3:** Summary of the VIKING Study

Full Title {1}	Suprachoroidal viscobuckling for the treatment of rhegmatogenous retinal detachment: a randomized, controlled, feasibility trial
Short Title {1}	VIKING study
Research Ethics Committee Number {24}	21/EM/0088
Clinicaltrial.Gov ID No {2a and 2b}	NCT04557527
IRAS ID {4}	283518
Sponsor {5b}	King’s College London and King’s College Hospital The R&I Office | First Floor Coldharbour Works | 245A Coldharbour Lane | Brixton | London SW9 8RR Tel: 020 3299 1980 Email: kch-tr.research@nhs.net
Funding {4}	BEAVRS and Norfolk and Norwich University Hospital Retina research fund 367
Role of Sponsor and Funder {5c}	The sponsor is responsible for study oversight and is the lead study site. The sponsor site’s study team (employed by the sponsor) is responsible for their site-level study data collection and management. The study funders are not responsible for the overall study design, data collection and management, analysis or interpretation, writing of this and subsequent reports, or decision to submit reports for publication
Protocol {3}	Version 1.2 10/10/21
Study Contributors and their roles {5a}	Timothy L. Jackson: Chief Investigator [CI] (concept and study design) Shohista Saidkasimova: Co-Chief Investigator (concept and study design) Chan Ning Lee: Co-investigator (protocol drafting) George S.P. Murphy: Co-investigator (protocol drafting)
Purpose	Primary objective: To establish if it is feasible to recruit, retain, and evaluate patients with retinal detachment into a larger randomised controlled trial of suprachoroidal viscobuckle (SCVB) vitrectomy Secondary objective: To make a preliminary assessment of safety and efficacy of SCVB vitrectomy
Study Design	A randomised, controlled, feasibility trial using a CE-marked surgical device
Key Inclusion criteria/Study Population	Adults requiring surgery for the treatment of primary rhegmatogenous retinal detachment caused by a single break, or multiple breaks within one clock hour
Number of Participants	50
Intervention {11a}	Surgical treatment of retinal detachment with vitrectomy and SCVB with the licenced high viscosity cohesive preparations of hyaluronic acid OVD (HEALON5™ PRO OVD [Johnson&Johnson], OphteisBio [Rayner], and NuVisc™ PRO [BVI])
Comparator {11a}	Standard vitrectomy with cryotherapy, retinopexy and gas tamponade
Study Duration	24 months
Safety Measures {12}	Adverse events and serious adverse events
Primary Efficacy Measure {12}	Feasibility outcomes: - Screen failure rate - Rate of recruitment - Protocol compliance - Participant retention and discontinuation Main efficacy outcome: Attachment of the retina with no intravitreal tamponade present and no subretinal fluid which could spread, without the need for further surgery, at week 12 Key secondary efficacy outcome: ETDRS best-corrected visual acuity at week 24
Number of Study Sites {9}	6 National Health Service (NHS) University Hospitals

## Abbreviations

AE: Adverse effect
ADE: Adverse device effect
BEAVRS: British and Eire Association of Vitreoretinal Surgeons
EDC: Electronic data capture system
IME: Important Medical Events
NNUH: Norfolk and Norwich University Hospital
OVD: Ophthalmic viscosurgical device
RD: Retinal detachment
RRD: Rhegmatogenous retinal detachment
REC: Research Ethics Committee
RPE: Retinal pigment epithelium
SAE: Serious Adverse Event
SADE: Serious Adverse Device Effects
SCVB: Suprachoroidal viscobuckle
VIKING study: Suprachoroidal VIsco-bucKlING versus gas tamponade for the treatment of rhegmatogenous retinal detachment

## References

[1] Mitry D, Awan MA, Borooah S, Siddiqui MAR, Brogan K, Fleck BW, et al. Surgical outcome and risk stratification for primary retinal detachment repair: results from the Scottish Retinal Detachment study. Br J Ophthalmol. 2012;96(5):730–4. Available from: http://bjo.bmj.com/lookup/doi/10.1136/bjophthalmol-2011-300581. 10.1136/bjophthalmol-2011-300581 22257789

[2] Jackson TL, Donachie PHJ, Sallam A, Sparrow JM, Johnston RL. United Kingdom National Ophthalmology Database study of vitreoretinal surgery: report 3, retinal detachment. Ophthalmology. 2014;121(3):643–8. Available from: https://linkinghub.elsevier.com/retrieve/pii/S0161642013006155. 10.1016/j.ophtha.2013.07.015 23978624

[3] Jackson TL, Donachie PHJ, Sparrow JM, Johnston RL. United Kingdom National Ophthalmology Database study of vitreoretinal surgery: report 1; case mix, complications, and cataract. Eye. 2013;27(5):644–51. 23449509 10.1038/eye.2013.12 PMC3650265

[4] Mohamed YH, Ono K, Kinoshita H, Uematsu M, Tsuiki E, Fujikawa A, et al. Success rates of vitrectomy in treatment of rhegmatogenous retinal detachment. J Ophthalmol. 2016. 10.1155/2016/2193518. 27478632 10.1155/2016/2193518 PMC4961815

[5] Custodis E. [Treatment of retinal detachment by circumscribed diathermal coagulation and by scleral depression in the area of tear caused by imbedding of a plastic implant]. Klin Monbl Augenheilkd Augenarztl Fortbild. 1956;129(4):476–95. Available from: http://www.ncbi.nlm.nih.gov/pubmed/13386159. 13386159

[6] Fallico M, Alosi P, Reibaldi M, Longo A, Bonfiglio V, Avitabile T, et al. Scleral buckling: a review of clinical aspects and current concepts. J Clin Med. 2022. 10.3390/jcm11020314. 35054009 10.3390/jcm11020314 PMC8778378

[7] Tyagi M, Basu S. Glue-assisted retinopexy for rhegmatogenous retinal detachments (GuARD): a novel surgical technique for closing retinal breaks. Indian J Ophthalmol. 2019;67(5):677–80. Available from: http://www.ncbi.nlm.nih.gov/pubmed/31007238. 10.4103/ijo.IJO_1943_18 PMC6498943 31007238

[8] Wang Q, Zhao J, Xu Q, Han C, Hou B. Intraocular application of fibrin glue as an adjunct to pars plana vitrectomy for rhegmatogenous retinal detachment. Retina. 2020;40(4):718–24. Available from: http://www.ncbi.nlm.nih.gov/pubmed/31259805. 10.1097/IAE.0000000000002584 31259805

[9] Aydin E, Eris E, Kazanci L, Uyar OM. Reattachment of rhegmatogenous retinal detachment via fibrin tissue adhesive. Korean J Ophthalmol. 2021;35(3):173–8. Available from: http://ekjo.org/journal/view.php?doi=10.3341/kjo.2020.0020. 10.3341/kjo.2020.0020 PMC8200591 33596627

[10] Martínez-Castillo V, Zapata MA, Boixadera A, Fonollosa A, García-Arumí J. Pars plana vitrectomy, laser retinopexy, and aqueous tamponade for pseudophakic rhegmatogenous retinal detachment. Ophthalmology. 2007. 10.1016/j.ophtha.2006.07.037. 17056117 10.1016/j.ophtha.2006.07.037

[11] El Rayes EN, Oshima Y. Suprachoroidal buckling for retinal detachment. Retina. 2013;33(5):1073–5. Available from: https://journals.lww.com/00006982-201305000-00027. 10.1097/IAE.0b013e318287daa5 23612022

[12] Schwenn O, Dick HB, Krummenauer F, Christmann S, Vogel A, Pfeiffer N. Healon5 versus Viscoat during cataract surgery: intraocular pressure, laser flare and corneal changes. Graefes Arch Clin Exp Ophthalmol. 2000;238(10):861–7. Available from: http://www.ncbi.nlm.nih.gov/pubmed/11127574. 10.1007/s004170000192 11127574

[13] El Rayes EN, Mikhail M, El Cheweiky H, Elsawah K, Maia A. Suprachoroidal buckling for the management of rhegmatogenous retinal detachments secondary to peripheral retinal breaks. Retina. 2017;37(4):622–9. Available from: http://journals.lww.com/00006982-201704000-00003. 10.1097/IAE.0000000000001214 27482642

[14] Szurman P, Boden K, Januschowski K. Suprachoroidal hydrogel buckling as a surgical treatment of retinal detachment: biocompatibility and first experiences. Retina. 2016;36(9):1786–90. 27429389 10.1097/IAE.0000000000001116

[15] Mikhail M, El-Rayes EN, Kojima K, Ajlan R, Rezende F. Catheter-guided suprachoroidal buckling of rhegmatogenous retinal detachments secondary to peripheral retinal breaks. Graefe’s Arch Clin Exp Ophthalmol. 2017;255(1):17–23. Available from: http://link.springer.com/10.1007/s00417-016-3530-8. 10.1007/s00417-016-3530-8 27853956

[16] Poole TA, Sudarsky RD. Suprachoroidal implantation for the treatment of retinal detachment. Ophthalmology. 1986;93(11):1408–12. Available from: https://linkinghub.elsevier.com/retrieve/pii/S016164208633553X. 10.1016/s0161-6420(86)33553-x 3808600

[17] Boden K, Januschowski K, Szurman P. Suprachoroidal hydrogel buckling. Ophthalmologe. 2018;115(11):967–71. 30120537 10.1007/s00347-018-0771-4

[18] Eldridge SM, Chan CL, Campbell MJ, Bond CM, Hopewell S, Thabane L, et al. CONSORT 2010 statement: extension to randomised pilot and feasibility trials. BMJ. 2016;i5239. Available from: https://www.bmj.com/lookup/doi/10.1136/bmj.i5239. 10.1136/bmj.i5239 PMC5076380 27777223

[19] Chan A-W, Tetzlaff JM, Altman DG, Laupacis A, Gøtzsche PC, Krleža-Jerić K, et al. SPIRIT 2013 statement: defining standard protocol items for clinical trials. Ann Intern Med. 2013;158(3):200. Available from: http://annals.org/article.aspx?doi=10.7326/0003-4819-158-3-201302050-00583. 10.7326/0003-4819-158-3-201302050-00583 PMC5114123 23295957

[20] El-Abiary M, Shams F, Goudie C, Yorston D. The Scottish RD survey 10 years on: the increasing incidence of retinal detachments. Eye. 2023;37(7):1320–4. Available from: https://www.nature.com/articles/s41433-022-02123-1. 10.1038/s41433-022-02123-1 PMC9159045 35650324

[21] Manufacturers guide to The HEALON5 PRO Ophthalmic Viscoelastic Device (OVD). https://www.jnjvisionpro.com/healon-pro.

[22] Julious SA. Sample size of 12 per group rule of thumb for a pilot study. Pharm Stat. 2005;4(4):287–91. Available from: https://onlinelibrary.wiley.com/doi/10.1002/pst.185.

[23] Sim J, Lewis M. The size of a pilot study for a clinical trial should be calculated in relation to considerations of precision and efficiency. J Clin Epidemiol. 2012;65(3):301–8. Available from: https://linkinghub.elsevier.com/retrieve/pii/S0895435611002642. 10.1016/j.jclinepi.2011.07.011 22169081

