# Supplementary material for: Suprachoroidal VIsco-bucKlING versus gas tamponade for the treatment of rhegmatogenous retinal detachment (VIKING): study protocol for a multi-centre, randomised, controlled feasibility study
Source: Pilot Feasibility Stud. 2026 Mar 17;12:78. doi: 10.1186/s40814-026-01787-w (PMC13188390; doi:10.1186/s40814-026-01787-w)
Supplement: Supplementary file 2 — Supplementary Material 2. [file 40814_2026_1787_MOESM2_ESM.docx]

Patient Consent Form for participation in **VIKING study**

(Suprachoroidal VIsco-bucKlING for the treatment of rhegmatogenous retinal detachment: a randomized, controlled, feasibility trial)

**IRAS Number 283518**

**Chief Investigator:** Professor Tim Jackson, Ophthalmology Dept, King’s College Hospital, London SE5 9RS

**Patient Identification Number for this trial:**

|  |  | | **Please initial box** |
| --- | --- | --- | --- |
| **1** | I confirm that I have read the information sheet dated 10/10/21 v3 for the above study. I have had the opportunity to consider the information, ask questions and have had these answered satisfactorily. | |  |
| **2** | I understand that my participation is voluntary and that I am free to withdraw at any time without giving any reason, without my medical care or legal rights being affected. | |  |
| **3** | I understand that relevant sections of my medical notes and data collected during the study, may be looked at by individuals from the sponsor of the trial (King’s College Hospital NHS Trust) and responsible persons authorized by the sponsor, from regulatory authorities or from the NHS Trust, where it is relevant to my taking part in this research. I give permission for these individuals to have access to my records. | |  |
| **4** | I understand that the information collected about me will be used to support other research in the future and may be shared anonymously with other researchers. | |  |
| **5** | I agree to my General Practitioner being informed of my participation in the study | |  |
| **6** | I agree to be contacted about ethically approved future research (optional) | |  |
|  | |  |  |
| **7.** | I agree to take part in the above study. | |  |

Name of Participant Date Signature

Name of Person Date Signature

taking consent

When completed: 1 for participant; 1 for researcher site file; 1 (original) to be kept in medical notes.
