# Supplementary material for: Suprachoroidal VIsco-bucKlING versus gas tamponade for the treatment of rhegmatogenous retinal detachment (VIKING): study protocol for a multi-centre, randomised, controlled feasibility study
Source: Pilot Feasibility Stud. 2026 Mar 17;12:78. doi: 10.1186/s40814-026-01787-w (PMC13188390; doi:10.1186/s40814-026-01787-w)
Supplement: Supplementary file 3 — Supplementary Material 3. [file 40814_2026_1787_MOESM3_ESM.docx]

Patient/ information sheet

**VIKING Study**

**(**SUPRACHOROIDAL **VI**SCO-BUC**K**L**ING** FOR THE TREATMENT OF RHEGMATOGENOUS RETINAL DETACHMENT**)**

**PART 1**

We would like to invite you to take part in our research study. Joining the study is entirely up to you. Before you decide we would like you to understand why the research is being done and what it would involve for you. One of our team will go through this information sheet with you, to help you decide whether you would like to take part and answer any questions you may have. We suggest this should take about 15 minutes and talk to others about the study if you wish.

The first part of the Participant Information Sheet tells you the purpose of the study and what will happen to you if you take part.

The second part will give you more detailed information about the conduct of the study. We will be happy to explain further if anything is unclear.

### **What is the purpose of the study?**

1. The purpose of the study is to assess the new treatment's safety before considering a bigger study.
2. The study explores a new way of treating retinal detachment by comparing it to the traditional way of treating the detachment with long-acting gas that normally takes 4-6 weeks to dissolve. In the new way of treating, we will use a viscobuckle (a gel-like substance (Sodium hyaluronate) which is injected under the retina) to help close the retinal hole that has caused your detachment instead of using gas.

The study is asking whether it is possible to run a larger trial comparing standard surgery with gas to surgery with viscobuckle without gas. The overall aim of undertaking the study is to improve patient’s outcome with faster recovery from surgery.

### **Why have I been invited?**

You have been invited to take part in the study because you have a medical condition called retinal detachment.

Patients from King’s College Hospital (KCH), Tennent Institute of Ophthalmology (Glasgow), St Thomas’ Hospital, Moorfields Eye Hospital, Sheffield University Hospital, Southend Hospital and Sunderland Eye Hospital will be invited to take part. We expect approximately 50 patients will take part.

1. **What is a retinal detachment?**
2. The retina is the light-sensitive layer of nerve cells lining the cavity of the eyeball and retinal detachment is a condition where the retina peels away from the wall of the eye. In most cases, the retina detaches because a hole or a tear has formed in the retina allowing fluid to pass underneath the retina. Most retinal detachments occur because of ageing process in the eye, but certain people are at higher risk than others. These include people who are short-sighted, those who have had cataract surgery in the past and those who have suffered a severe eye injury. Some types of retinal detachments can run in families, but these are rare.
3. **Do I have to take part?**
4. It is up to you to decide. We will describe the study and go through this information sheet, which we will then give to you. You will be able to keep this information sheet and think about taking part. You are free to discuss the information with anyone you wish including your family and friends. If you agree, we will then ask you to sign a consent form to show you have agreed to take part. You are free to withdraw at any time, without giving a reason. This would not affect the standard of care you receive.

### **What will happen to me if I take part?**

1. Consent

You will be asked to sign written consent only after you have been fully informed and wish to take part in the study. We will answer any of the questions you may have before consenting to take part in this study.

1. Screening

You are eligible to be considered to take part in this study if you have been diagnosed with a new retinal detachment due to one or two retinal breaks. The final decision on your eligibility will occur during the operation once the full internal eye examination is completed.

1. Treatment

You will receive the surgery to treat the retinal detachment which you have developed. The most commonly used surgery for retinal detachment, which we will refer to as “standard treatment”, is vitrectomy (ie removal of jelly inside the eye), cryotherapy (use of freezing treatment around the retinal break that caused the retinal detachment to aid its healing) and insertion of a gas bubble (to keep the retina in place whilst its healing). The less commonly used treatment is scleral buckling surgery with a silicon plastic tube placed on the outer layer of the eyeball (sclera) to push the wall of the eyeball towards the retinal break.

In this study we will be using ‘viscobuckle’ which is created by a viscous gel, which is widely used during eye surgery. The study compares standard treatment with a surgical variation where instead of gas we use an injection of thick gel (viscoelastic) underneath the retinal tear. The advantage of a viscobuckle is that it only lasts 7-10 days, long enough time to induce healing of retinal break and is thereafter absorbed.

We will decide at the time of surgery to treat you either with standard treatment using vitrectomy and gas or the new treatment with vitrectomy and temporary viscobuckle without gas inside the eye. You will be randomly allocated to either standard surgery or the new technique, with an equal chance of each option. This process of ‘randomisation’ is undertaken by a computer and ensures that we allocate patients to each option in a fair and unbiased way.

Both procedures can be carried out under local or general anaesthesia and will take a similar amount of time (30-60 minutes depending on the complexity of your detachment).

Other than this variation in surgical technique, post-operative review and management reflects standard care.

1. Follow up

After the surgery, you will be followed up under a typical standard of care, on day 1, and weeks 1-2, 4-6, 12 and 24. We will follow you up for 6 months in total.

**What are the alternatives for treatment?**

The most common treatment for retinal detachment is vitrectomy with cryotherapy and intraocular gas surgery or a scleral buckle (plastic tube placed outside retinal tear and detachment area) procedure for certain types of detachment.

**What are the possible benefits of taking part?**

**RD surgery is mostly an effective treatment. However, it imposes a material burden on patients due to gas tamponade. Our proposed surgical treatment is focused on improving patient’s experience and recovery.** By eliminating the use of a long-acting intraocular gas patient’s post-operative recovery should be easier and faster, as gas tamponade causes very blurred vision for a month or two, and usually requires head positioning, for example, left cheek to a pillow for 50 minutes out of every hour for a week after surgery. Avoiding the use of gas may also slow the onset of cataract that occurs after retinal detachment surgery and reduce the risk of problems with pressure inside the eyeball after surgery.

**Therefore, a surgery that avoids or minimises the use of long-acting gas would speed the patient's recovery, avoid the need for positioning and allow earlier return to normal activities including driving and flying. It will also benefit society if patients can return to work sooner, and not be as dependent on others during their convalescence.**

We do not know if it might have a lower or higher chance of success than standard surgery and we cannot promise the study will help you but the information we get from this study will help improve the future treatment of people with retinal detachment.

**What are the possible disadvantages and risks of taking part?**

Both types of retinal detachment surgery are subject to risks.

The risks associated with standard surgery include failure of surgery (approximately 15%), severe infection 1:1000, severe bleeding 1:1000, loss of sight, cataract is very common and develops in a majority of patients requiring further surgery (unless you have previously had cataract surgery), increased pressure in the eyeball (is usually transient and responds to additional drops). All these risks will also apply to the new variant of surgery.

The risks applied specifically to standard surgery with gas is the slow resolution of gas which may take up to 6 weeks to dissolve and in that time, you are not allowed to fly, advised not to drive and may not be suitable for a certain type of general anaesthesia.

The risks of no gas treatment with viscobuckle include allergic reaction to the viscous substance- sodium hyaluronate gel. However, it is a licenced substance used in eye surgery and such reactions are extremely rare. If you develop any pain, more than would be expected from a normal postoperative course, we will ask you to contact your surgeon or the emergency team at your earliest convenience (eye casualty nursing team or on call doctor).

Although not reported, it is possible that as a new technique, the success rate with viscobuckle vitrectomy is lower than standard surgery, with a greater risk of retinal redetachment. There may also be cases of technical failure, wherein it is not possible to deliver the gel as required, and you will require gas tamponade.

You may develop a small haemorrhage at the site of surgery without any long-term effects (i.e. retinal or choroidal haemorrhage). It is also possible that the substance may move from where it was intended (migration of gel into the subretinal space) which we expect to resolve without further treatment.

We do not anticipate any different impact on insurance, pregnancy, or breastfeeding.

**Who is organising and funding this study?**

King’s College London will be the sponsor, and King’s College Hospital will be the co-sponsor of the study. The doctors in charge of this study are Prof Tim Jackson (KCH) and Dr Shohista Saidkasimova (Glasgow).

The study is funded by a grant from the British Vitreoretinal Society and Retina Research Fund from Norfolk and Norwich University Hospital (NNUH).

Your hospital doctor is not paid for including you in this study.

**How have patients and the public been involved in this study?**

The proposed study was discussed with members of the public and patients who have received such treatment and were involved in reviewing the Participant Information Sheet.

**Who has reviewed this study?**

All research in the NHS is looked at by an independent group of people, called a Research Ethics Committee, to protect your interests. This study has been reviewed by Leicester Central Research Ethics Committee. It has been approved by the Health Research Authority and each local hospital will confirm that the study can go ahead.

You may be contacted about ethically approved future research.

**Expenses and Payments**

There are no funds available for payments to those participating in this study. However, as part of standard treatment, our patients are eligible for hospital transport and voluntary community car service where necessary.

**What happens when the research study stops?**

This study will continue for 24 months. You will receive full treatment with five follow up appointments over 6 months. After this, you will be discharged back to the care of your community optometrist. If you have any problems, we will be able to address them as part of your regular NHS treatment.

**This completes Part 1 of the Information Sheet.**

If the information in Part 1 has interested you and you are considering participation, please continue to read the additional information in Part 2 before making any decision.

### **PART 2**

### **What if new information becomes available?**

Sometimes we get new information about the treatment being studied. If this happens, your study doctor will tell you and discuss whether you should continue in the study. If you decide not to carry on, your study doctor will continue your care. If you decide to continue in the study, he/she may ask you to sign an agreement outlining the discussion*.*

This new information that becomes available might specifically affect you and your health. If this happens, your study doctor might consider that you should withdraw from the study. He/she will explain the reasons for withdrawing from the study and arrange for your care to continue*.*

If the study is stopped for any other reason, we will tell you and arrange for your continuing care.

1. **What will happen if I do not want to carry on with the study?**

Your decision to withdraw from the study will not affect the care you receive unless you withdraw before surgery. In that case, you will not receive the new surgical technique, but standard surgery instead. Information already collected about you will be used even if you leave the study or withdraw your consent, but no further information will be collected. You will be entitled to standard NHS treatment after withdrawing from the study.

1. **What if there is a problem?**

If you have a concern about any aspect of this study, you should ask to speak to your study doctor who will do their best to answer your questions {insert name and contact number}. If you remain unhappy and wish to complain formally, you can do this through the NHS Complaints procedure by contacting your local Patient Advice Liaison Service (PALS) office ([What is PALS (Patient Advice and Liaison Service)? - NHS (www.nhs.uk.)](https://www.nhs.uk/nhs-services/hospitals/what-is-pals-patient-advice-and-liaison-service/)). Details of your local office can be obtained by asking your study doctor or nurse, or looking on the NHS website (Find Patient advice and liaison services (PALS) services - NHS (www.nhs.uk))

Every care will be taken during this study, however, in the unlikely event that you are injured by taking part, compensation may be available.

If something does go wrong, and you are harmed during the research and this is due to someone's negligence then you may have grounds for legal action for compensation against your NHS Trust but you may have to pay your legal costs.

Regardless of this, if you wish to complain, or have any concerns about any aspect of the way you have been approached or treated by members of staff or about any side effects (adverse events) you may have experienced due to your participation in the study, the normal NHS complaints mechanisms are available to you. Please ask your study doctor if you would like more information on this.

1. **Will my taking part be kept confidential?**
2. We will keep information about you confidential and secure.
3. Your name will not be used in any reports about the study. Your data will be stored in accordance with the principle of the Data Protection Act 2018 and relevant trial governance regulations.

Paper trial documents will be stored in secure, restricted access, research offices. Core clinical data will be available in the NHS electronic medical records, or NHS paper documents, which includes personal information, but once extracted these will be relabelled using an anonymised trial identifier.

Data generated from the study will be de-identified using a unique study number. Only de-identified data will be transferred between hospitals and will use password-protected files.

Your surgeon will have access to your medical notes to decide if you are eligible to participate in the first instance and to confirm eligibility after informed consent has been given. Research staff will also need to record personal data such as medical history and adverse events.

If you consent to take part in the research, any of the information collected about you may be inspected by the sponsor (including representatives of the sponsor). These inspections are solely for the research and analysing the results. Your records may also be looked at by the regulatory authorities or ethics committees to check that the study is being carried out correctly. However, all such activities will occur with your consent and under the direct control and supervision of your surgeon.

1. Involvement of the General Practitioner/Family Doctor (GP)
2. We will need to write to your GP to keep them informed of your treatment. With your consent, your GP will be informed of your involvement in the trial. Any other medical practitioners who treat you, e.g. should you be admitted to the hospital for any reason, will also be informed.
   - 1. **What will happen to the results of the research study?**

We intend to publish our research findings in medical journals. If you wish to be informed of the outcome of the study, we will be happy to provide a copy of the published report and your study doctor or attending eye doctor will be able to explain its contents. The information collected during this study will be used to support other research in the future and maybe shared anonymously with other researchers.

**How we will use your data**

We will need to use information from your medical records for this research project.

This information will include your initials, hospital number and age.

People will use this information to do the research or to check your records to make sure that the research is being done properly.

We will keep all information about you safe and secure in a secure database.

Once we have finished the study, we will keep some of the data so we can check the results. We will write our reports in a way that no one can work out that you took part in the study.

**What are your choices about how your information is used?**

You can stop being part of the study at any time, without giving a reason, but we will keep information about you that we already have.

- If you choose to stop taking part in the study, we would like to continue collecting information about your health from your hospital records. If you do not want this to happen, tell us and we will stop.
- We need to manage your records in specific ways for the research to be reliable. This means that we won’t be able to let you see or change the data we hold about you.

**Where can you find out more about how your information is used?**

You can find out more about how we use your information:

- on the Health Research Authority website [www.hra.nhs.uk/information-about-patients/](https://www.hra.nhs.uk/information-about-patients/)
- in a leaflet called: HowWeWillUseYourData KCH V1 (21-11-19) – available from the study team
- at our website <https://www.kch.nhs.uk/about/corporate/data-protection>
- by emailing our Data Protection Officer on [kch-tr.dpo@nhs.net](mailto:kch-tr.dpo@nhs.net)

1. **Thank you** for considering taking part and taking the time to read this information sheet.
2. If you decide to take part in the study, we will give you a copy of the information sheet and a signed consent form to keep.

**Further information about retinal detachment and its standard treatment can be found on-line:**

[http://beavrs.org/retinal-detachment-surgery#](http://beavrs.org/retinal-detachment-surgery) and <https://www.rnib.org.uk/sites/default/files/Understanding_Retinal_Detachment_NV.pdf>

**For information on taking part in research please contact:** INVOLVE, Alpha House, University of Southampton Science Park, Chilworth, Southampton, SO16 7NS Telephone: 023 8059 5628, Email: [involve@nihr.ac.uk](mailto:involve@nihr.ac.uk)

Local Contacts:

Your doctor ........................................................... Tel: ......................................................

Your nurse/study coordinator........................................... Tel: ........................................................
